# Secondary Solid Organ Neoplasm in Patients with Acute Lymphoblastic Leukemia: A Nationwide Population-Based Study in Taiwan

**DOI:** 10.1371/journal.pone.0152909

**Published:** 2016-04-01

**Authors:** Chung-Jen Teng, Leh-Kiong Huon, Yu-Wen Hu, Chiu-Mei Yeh, Sheng-Hsuan Chien, San-Chi Chen, Chia-Jen Liu

**Affiliations:** 1 Division of Hematology and Oncology, Department of Medicine, Far Eastern Memorial Hospital, Taipei, Taiwan; 2 School of Medicine, National Yang-Ming University, Taipei, Taiwan; 3 Department of Otolaryngology-Head & Neck Surgery, Cathay General Hospital, Taipei, Taiwan; 4 School of Medicine, Fu Jen Catholic University, Taipei, Taiwan; 5 Cancer Center, Taipei Veterans General Hospital, Taipei, Taiwan; 6 Department of Family Medicine, Taipei Veterans General Hospital, Taipei, Taiwan; 7 Division of Hematology and Oncology, Department of Medicine, Taipei Veterans General Hospital, Taipei, Taiwan; 8 Institute of Clinical Medicine, National Yang-Ming University, Taipei, Taiwan; 9 Institute of Public Health, National Yang-Ming University, Taipei, Taiwan; Queen's University Belfast, UNITED KINGDOM

## Abstract

**Background:**

Acute lymphoblastic leukemia (ALL) is more common in children than in adults. Secondary neoplasms (SNs) in childhood ALL have been widely reported. However, only one study has demonstrated SNs in adult ALL. Because of the poorer survival of adult ALL, the incidence might be underestimated.

**Objective:**

To evaluate the incidence and risk factors of secondary solid organ neoplasms among adult and child ALL patients.

**Methods:**

Newly diagnosed ALL patients between 1997 and 2011 were recruited from the Taiwan National Health Insurance database. Those who had antecedent or combined malignancies were excluded. Standardized incidence ratios (SIRs) were analyzed to compare the risk of our cohort to general population in the same age, sex and calendar year. Risk factors for SN development were analyzed by Cox proportional hazards models. Effects of treatments were treated as time-dependent variables.

**Results:**

The 15-year cumulative incidence of SN was 1.9% and 8.4% in 1,381 child and 2,154 adult ALL patients, respectively. The SIR was significantly increased in child ALL (SIR 6.06), but not in adult ALL (SIR 1.16). The SIRs of follow-up periods were 5.14, 2.24, .87 and .71 at ≥ 10 years, 5–10 years, 1–5 years and 0–1, respectively. Overall, 15 SNs developed, and CNS tumors (SIR 11.56) were the most common type. Multivariate analysis showed that age ≥ 20 years (hazard ratio [HR] 5.04), end-stage renal disease (HR 18.98) and cranial irradiation (HR 8.12) were independent risk factors for cancer development.

**Conclusions:**

When compared with the general population, child ALL shows a increased risk of developing SNs. CNS tumors are the most common type, and cranial irradiation is an independent risk factor. With longer follow-up, the risk of SNs increases. Hence, physicians need to pay more attention on the risk of developing SNs in long-term ALL survivors with risk factors.

## Introduction

Acute lymphoblastic leukemia (ALL) accounts for about 25% of all childhood cancers, and is the most common leukemia in children [1], but represents less than 20% of adult acute leukemia. The survival for adult and especially child ALL has improved over the past decades with risk-directed combination chemotherapy, including central nervous system (CNS) prophylaxis[2]. With the increased survival of ALL, long-term complications have been observed [3, 4], such as anthracycline-induced cardiomyopathy [5], cranial irradiation-induced neurocognitive deficits [6] and endocrine abnormalities [7]. Apart from these late sequelae, secondary neoplasms (SNs) are a serious complication for ALL survivors.

Of these SNs in child ALL, hematologic malignancies [8–10] and CNS tumors [11–14] are most commonly reported. In some studies, skin tumors are common as well [4, 9]. These variations may be due to the different treatment strategies and ethnicity. For example, cranial RT has been especially associated with CNS tumors, with a dose-dependent effect [11, 13]. In regards to ethnicity, the epidemiology of cancer varies in different areas, which may have an influence on the types of SNs in ALL patients. All the previous study cohorts were in the Unites States and Europe, but no population-based cohort in Asia has been reported. On the other hand, the rarity and poorer prognosis of adult ALL makes it difficult to conduct a large population-based study as significant as those already conducted on child ALL. Most of the studies have focused on child ALL, and only one study examined SNs in adult ALL [15]. Hence, it is worthwhile to conduct a nationwide population-based cohort study in Asia, including both child and adult ALL.

The advantages of Taiwan’s National Health Insurance Research Dataset (NHIRD) are that it owns a nationwide population dataset of all registered malignancies, with a long observation period. The aim of this study is to use NHIRD to analyze the incidence and types of secondary solid organ neoplasm among both child and adult ALL patients and to identify the potential risk factors, including chemotherapy, radiotherapy and comorbidities.

## Materials and Methods

### Data Sources

Taiwan's National Health Insurance (NHI) program, which began in 1995, is a mandatory universal health insurance program. It supplies comprehensive medical care to all Taiwan’s residents, with a coverage rate of over 99% [16]. Medical coverage includes outpatient, inpatient, emergency, dental, and traditional Chinese medicine services.

NHIRD is managed by Taiwan’s National Health Research Institute. In addition, catastrophic illness data logged in the NHI database provides comprehensive information on the enrollment and health care resources of all patients with severe diseases. Those patients who meet the criteria of “catastrophic illness” have copayment exemptions under the NHI program. So we have integrated several NHI databases, including claims data, NHI enrollment files and the registry for drug prescriptions. Acute lymphoblastic leukemia and all the other malignancies are categorized as catastrophic illnesses. All information that might identify an individual patient is encrypted, so that this research was exempted from informed consent. The data is confidential, as mandated by the Bureau of National Health Insurance and the National Health Research Institutes. This study has been reviewed by the institutional review board of Taipei Veterans General Hospital that is an appropriated regulatory agency to review researches both in adults and children. (VGHTPE-IRB: 2013-10-002CE).

### Study Population

Our nationwide population-based cohort study enrolled newly diagnosed ALL patients who were retrieved from the Registry of Catastrophic Illness from January 1, 1997 to December 31, 2011. Diagnosis was based on the International Classification of Diseases, Ninth Revision, Clinical Modification (ICD-9-CM) code 204. Histological evidence for ALL diagnosis is required for subjects identified from the Registry of Catastrophic Illness. ALL patients under 20 years of age were categorized as child ALL, and those aged above 20 years was described as adult ALL. We excluded those who had antecedent or combined other malignancies. Data on comorbidities and ALL treatment were collected for further analysis. Comorbidities of chronic pulmonary disease included chronic bronchitis, emphysema, asthma and chronic obstructive pulmonary disease.

In Taiwan, most child ALL were treated with Taiwan Pediatric Oncology Group (TPOG) protocol (TPOG-ALL-97 and TPOG-ALL-2002), which included steroids, vincristine, anthracycline, L-asparaginase, cyclophosphamide, cytarabine and 6-mercaptopurine in induction and re-induction; methotrexate, 6-mercaptopurine in consolidation and maintenance. Adult ALL patients in Taiwan were treated according to the chemotherapy principles of National Comprehensive Cancer Network (NCCN) Clinical Practice Guidelines in Oncology. Generally the chemotherapy drugs include cyclophosphamide, daunorubicin/doxorubicin, vincristine, prednisone/dexamethasone, aspargase, 6-mercaptopurine, alternating with high-dose methotrexate-cytarabine, with or without rituximab for CD20-positive disease.[17–20] Hematopoietic stem-cell transplantation (HSCT), usually having total body irradiation in conditioning regimen, was reserved for high risk or refractory/relapse disease.

### Outcome Measure and Diagnosis of Secondary Solid Organ Neoplasms

The general follow-up strategy in the whole cohort adhered to the suggestions for ALL in international treatment guidelines. Briefly, the post-consolidation surveillance included a thorough physical examination, complete blood test with differential, liver and renal function tests at least every 1–2 months in year 1 and every 3 months after year 2. A further examination would be initiated in case of unusual subjective complaints. The main dependent variable was the development of secondary solid organ neoplasms. A pathologic confirmation, which was subject to periodic review by the NHI Bureau, was mandatory for all neoplasms except hepatocellular carcinomas. We followed these patients until the occurrence of secondary solid organ neoplasms, death, or dropout from the NHI program, before the end of the year 2011.

### Statistical Analysis

Standardized incidence ratio (SIR) is defined as the observed number of cancer occurrences divided by the expected number, to determine the risk of secondary neoplasms among this study cohort. To count the expected numbers of cancers, the cancer incidence rate in the general population is multiplied by age, sex and calendar year in five-year intervals by the corresponding stratum-specific person-time accrued in the cohort. The cancer incidence rate among the general population was obtained from Taiwan’s National Cancer Registry. According to the assumption that the observed numbers of cancers followed a Poisson probability distribution, the 95% confidence intervals (CIs) of the SIRs were estimated. We defined the SIRs for subgroups based on sex, age, and period of time developing secondary solid organ neoplasms. In addition, the SIRs for different types of cancers were estimated.

To assess the incidence of developing secondary neoplasms during the 15-year observation period, the Kaplan-Meier method was employed. By using univariate and multivariate Cox proportional hazards models, we analyzed the risk factors for developing secondary solid organ neoplasms, such as age, sex, comorbidities, chemotherapy, radiotherapy and hematopoietic stem cell transplantation. The factors of ALL treatment were treated as time-dependent variables to prevent immortal time bias.[21] Those factors with *p* value < .1 in univariate analysis were entered into Cox multivariate analysis.

Data were extracted and computed using the Perl programming language (version 5.12.2; Perl Foundation, Walnut, CA). Data linkage, processing, and sampling were conducted by using Microsoft SQL Server 2008 (Microsoft Corp., Redmond, WA). SAS 9.2 software (SAS Institute Inc., Cary, NC) was used for all statistical analysis. Statistical significance was defined as a *p* value of < .05.

## Results

### Characteristics of the Study Population

**Fig 1:**
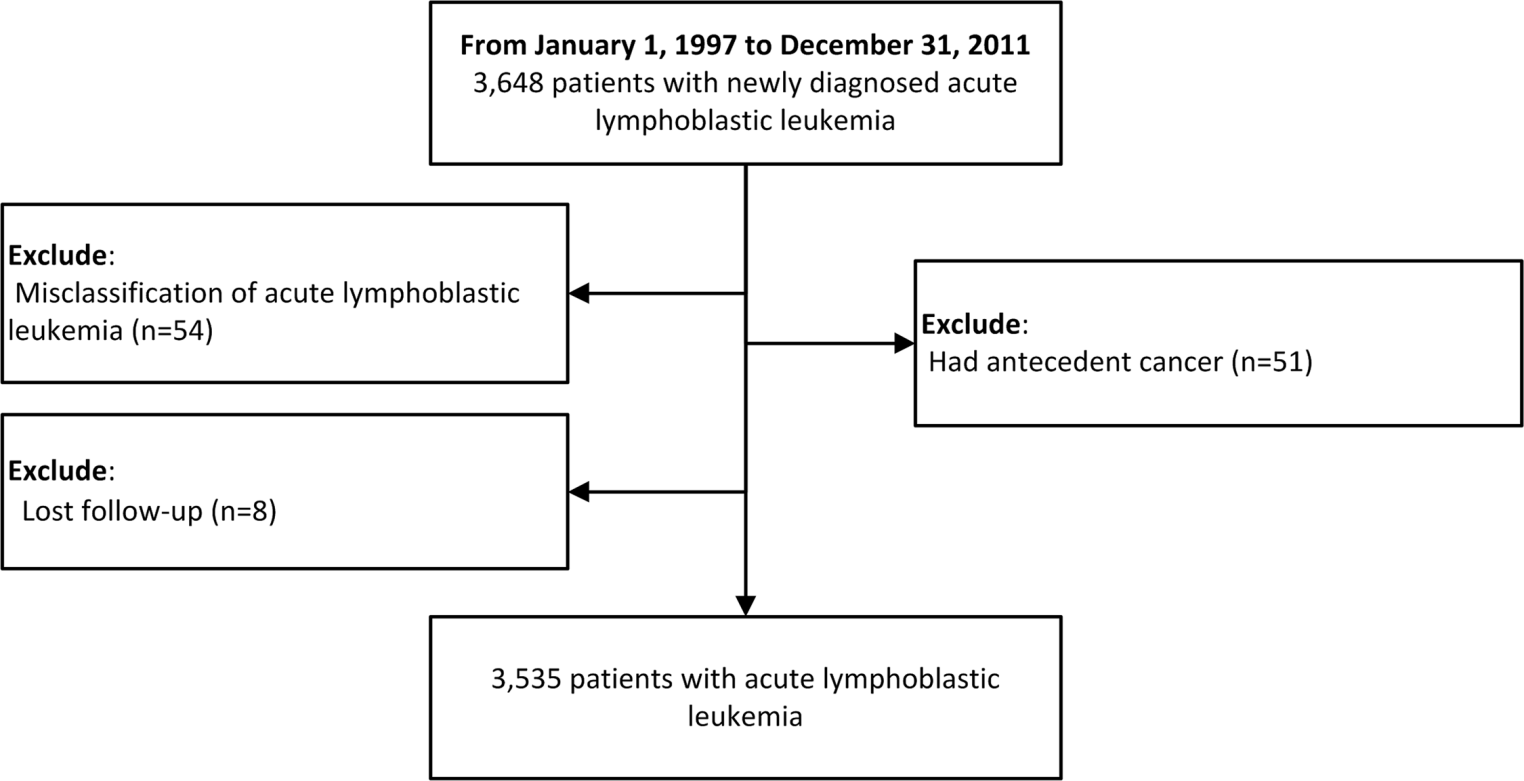
Flow chart.

We identified 3,648 patients with acute lymphoblastic leukemia in NHIRD’s catastrophic illness registry from 1997 to 2011. Of these, 54 patients were misclassified, 51 patients had antecedent malignancies before ALL, and eight patients were lost to follow-up. The flow chart is presented in Fig 1. The final sample consisted of 3,535 patients, including 2,018 (57%) males and 1,517 (43%) females, of a median age of 14 years at diagnosis (interquartile range, 5–35 years). According to the age, 2,154 (61%) had child ALL and 1,381 (39%) had adult ALL. Overall, this cohort was observed for 16,402 person-years from 1997 to 2011. Comorbidities and treatment are also listed. Among these patients, 858 (24.3%) received cranial irradiation, 360 (10.2%) total body irradiation (TBI), and 508 (14.4%) hematopoietic stem cell transplantation. The characteristics are shown in Table 1.

**Table 1:** Characteristics of patients with acute lymphoblastic leukemia.

	Total	Adult (age ≥ 20)	Child (age < 20)
No. of patients	3,532	1,379	2,154
Person-years at risk	15,020.5	2,352.5	12,668.0
Median age, years (interquartile range)	14 (4–34)	43 (29–58)	6 (3–12)
Age at diagnosis, years			
0–19	2,153 (61.0)	0 (0.0)	2,153 (100.0)
20–39	619 (17.5)	619 (44.9)	0 (0.0)
≥ 40	760 (21.5)	760 (55.1)	0 (0.0)
**Comorbidities**			
Diabetes mellitus	235 (6.7)	224 (16.2)	11 (0.5)
Chronic pulmonary disease	280 (7.9)	215 (15.6)	65 (3.0)
ESRD	35 (1.0)	32 (2.3)	3 (0.1)
Cirrhosis	48 (1.4)	46 (3.3)	2 (0.1)
Autoimmune diseases	146 (4.1)	89 (6.5)	57 (2.7)
Dyslipidemia	247 (7.0)	235 (17.0)	12 (0.6)
**Treatment**			
Anthracyclines	3,147 (89.1)	1,114 (80.8)	2,033 (94.4)
Akylating agents	2,197 (62.2)	898 (65.1)	1,299 (60.3)
Antimetabolites	2,995 (84.8)	955 (69.3)	2,040 (94.8)
Topo-II inhibitor	1,443 (40.9)	424 (30.8)	1,019 (47.3)
Asparaginase	2,684 (76.0)	737 (53.4)	1,947 (90.4)
Cranial irradiation	858 (24.3)	294 (21.3)	564 (26.2)
TBI	360 (10.2)	202 (14.7)	158 (7.3)

Abbreviations: ESRD, end-stage renal disease; Topo-II, topoisomerase II; TBI, total body irradiation; HSCT, hematopoietic stem cell transplantation

### All Cancers

**Fig 2:**
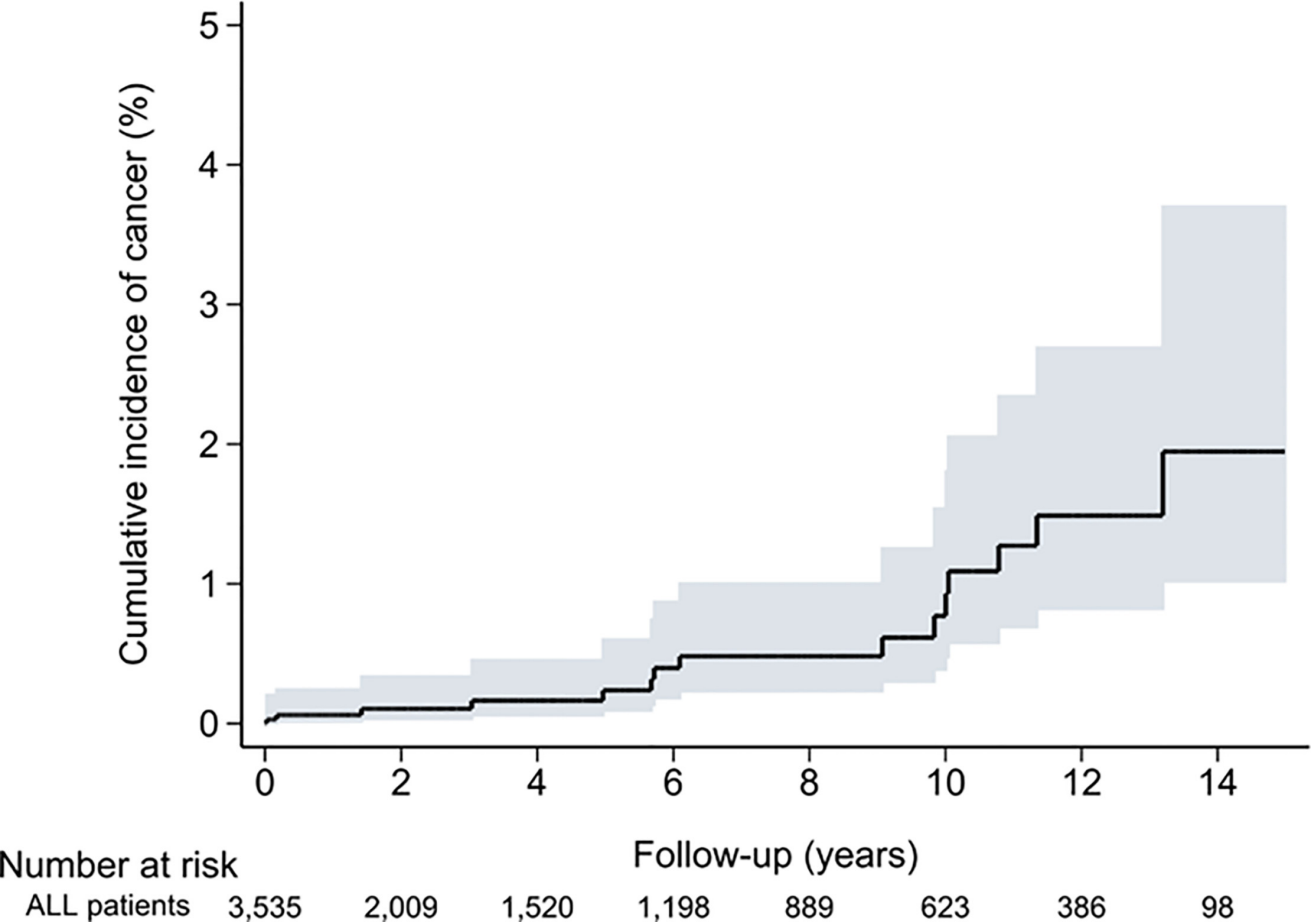
Cumulative incidence of second neoplasm in patients with acute lymphoblastic leukemia. Abbreviations: ALL, acute lymphoblastic leukemia.

During the follow-up period, a total of 15 patients, including seven males and eight females, developed secondary solid organ neoplasms. To compare with the general population, patients with ALL had an SIR of 1.58 (95% CI .50–2.06), and the trend was similar between male (SIR, 1.52; 95% CI, .61–3.12) and female (SIR, 1.65; 95% CI, .71–3.25) patients. In subgroup analysis, the SIRs were significantly increased in patients aged 0–19 (SIR, 6.06; 95% CI, 1.97–14.14) and 20–39 (SIR, 3.30; 95% CI, 1.07–7.70), but not in ≥ 40 (SIR, .60; 95% CI, .23–1.63), which means the risk was increased for child ALL but not for adult ALL (SIR, 1.16; 95% CI, .59–2.06). Regarding the follow-up period of ALL patients, a total of two, three, five and five patients developed SNs during the follow-up periods of 0–1, 1–5, 5–10 and ≥ 10 years, respectively. SIRs increased significantly only in the period of follow-up ≥ 10 years. These results are summarized in Table 2. Fig 2 shows the curve of cumulative incidence of developing SNs. Furthermore, the incidence of SNs were 8.4% in adult and 1.9% in child ALL patients (not shown in figure).

**Table 2:** Standardized incidence ratios according to sex, age at diagnosis and duration of acute lymphoblastic leukemia.

	Total			Male			Female		
Characteristics	Observed	Expected	SIR (95% CI)	Observed	Expected	SIR (95% CI)	Observed	Expected	SIR (95% CI)
All cancers	15	9.47	1.58 (.89–2.61)	7	4.62	1.52 (.61–3.12)	8	4.85	1.65 (.71–3.25)
Age at diagnosis, years									
0–19	5	.83	6.06 (1.97–14.14)	3	.48	6.27 (1.29–18.34)	2	.35	5.77 (.70–20.83)
20–39	5	1.51	3.30 (1.07–7.70)	3	.66	4.53 (.94–13.25)	2	.85	2.34 (.28–8.47)
≥ 40	5	7.14	.70 (.23–1.63)	1	3.47	.29 (.01–1.61)	4	3.65	1.10 (.30–2.81)
Follow-up time period after acute lymphoblastic leukemia									
0–1	2	2.81	.71 (.09–2.57)	0	1.49	.00 (.00–2.47)	2	1.32	1.51 (.18–5.47)
1–5	3	3.45	.87 (.18–2.54)	2	1.64	1.22 (.15–4.41)	1	1.82	.55 (.01–3.07)
5–10	5	2.24	2.24 (.73–5.22)	2	1.01	1.97 (.24–7.12)	3	1.22	2.46 (.51–7.18)
≥ 10	5	.97	5.14 (1.67–12.00)	3	.48	6.30 (1.30–18.42)	2	.50	4.03 (.49–14.56)

Abbreviations: SIR, standardized incidence ratio; CI, confidence interval

### Specific Cancer Types

These SNs included cancers of the CNS (5), breast (3), head and neck (2), esophagus (1), liver and biliary tract (1), lung and mediastinum (1), uterus (1) and bladder (1). Although almost all the SNs were of rare events and carried a non-significant SIR for SN development, the SIR (11.56; 95% CI, 3.75–26.97) of CNS tumors significantly increased. The results are listed in detail in Table 3. Among the five cases of CNS tumors, one underwent chemotherapy, one radiotherapy and one radiotherapy followed by chemotherapy. These three patients remained alive at the end of the year 2011, with a mean follow-up period of 3.0 years after the diagnosis of CNS tumors. The other two patients did not receive any cancer treatment. One remained alive with a follow-up period of 8 months and one died 7 months after the diagnosis of CNS tumors.

**Table 3:** Standardized incidence ratios for secondary neoplasms, according to cancer site.

	Total		
Site of cancer	Observed	Expected	SIR (95% CI)
All cancers	15	9.47	1.58 (.89–2.61)
Head and neck	2	1.04	1.91 (.23–6.91)
Esophagus	1	.17	5.74 (.15–32.01)
Liver and biliary tract	1	1.21	.82 (.02–4.59)
Lung and mediastinum	1	.92	1.09 (.03–6.09)
Breast	3	1.34	2.24 (.46–6.54)
Uterus	1	.20	4.98 (.13–27.76)
Bladder	1	.23	4.31 (.11–24.04)
CNS	5	.43	11.56 (3.75–26.97)

Abbreviations: SIR, standardized incidence ratio; CI, confidence interval; CNS, central nervous system

### Predictors of Cancer Risk

Univariate Cox proportional hazards analysis showed that being aged > 20, having chronic obstructive disease and end-stage renal disease (ESRD) were risk factors of SN development. Multivariate analysis showed an age of > 20 (HR 5.04, 95% CI 1.54–16.46, *p* = .007) and ESRD (HR 18.98, 95% CI 1.85–194.45, *p* = .013) remained independent predictors. When the specific treatment interventions were taken into consideration, cranial irradiation (HR 8.12, 95% CI 2.28–28.92, *p* = .001) was the independent risk factor for SN development by analyzing as a time-dependent covariate. The results are listed in detail in Table 4. After excluding patients who underwent HSCT, SIR remained significantly increased in follow-up period at > 10 years, and CNS tumor remained the most common type of neoplasms; end-stage renal disease (ESRD) and cranial irradiation remained independent risk factors for cancer development. (S1, S2 and S3 Tables) However, ESRD increased SNs risk significantly only in adult patients, but not in child. (S4 and S5 Tables)

**Table 4:** Risk factors for secondary neoplasm development in patients with acute lymphoblastic leukemia.

	Univariate analysis		Multivariate analysis^a^	
Variables	HR (95% CI)	*P* Value	HR (95% CI)	*P* Value
Age ≥ 20	7.57 (2.65–21.64)	< .001	5.04 (1.54–16.46)	.007
Sex (male)	.70 (.25–1.93)	.490	.69 (.25–1.92)	.476
**Comorbidities**				
Diabetes mellitus	†			
Chronic pulmonary disease	5.59 (1.21–25.88)	.028	3.23 (.65–16.13)	.154
ESRD	23.33 (2.96–184.02)	.003	18.98 (1.85–194.45)	.013
Cirrhosis	†			
Autoimmune diseases	†			
Dyslipidemia	†			
**Treatment**^b^				
Anthracyclines	.63 (.17–2.25)	.472		
Akylating agents	2.13 (.71–6.38)	.178		
Antimetabolites	1.01 (.22–4.57)	.990		
Topo-II inhibitor	.95 (.32–2.85)	.926		
Asparaginase	.41 (.14–1.19)	.100	.44 (.13–1.47)	.183
Cranial irradiation	4.72 (1.51–14.68)	.008	8.12 (2.28–28.92)	.001
TBI	4.35 (1.20–15.79)	.026	.57 (.09–3.64)	.555
HSCT	6.69 (2.19–20.42)	.001	3.99 (.78–20.31)	.096

Abbreviations: ESRD, end-stage renal disease; Topo, topoisomerase; TBI, total body irradiation; HSCT, hematopoietic stem cell transplantation.

^a^All factors with *p* < .1 in univariate analyses were included in the Cox multivariate analysis.

^b^Treatment was analyzed as a time-dependent covariate in the Cox regression model.

**†**: Don’t converge.

## Discussion

We present this first nationwide population-based study in Asia to demonstrate the risk of development of secondary solid organ neoplasms among both child and adult ALL patients. Our main findings include: (1) the risk of SN is significantly increased in childhood ALL (SIR 6.06) with a 15-year observation period; (2) the most common SN is CNS tumors; (3) cranial irradiation is an independent risk factor.

Our study demonstrates that the 15-year cumulative incidence of SNs is 8.4% in adult ALL and 1.9% in in child ALL, which is compatible with previous studies. The only one study about SN risk in adult ALL was reported by Tavernier *et al*, which showed the cumulative incidence of 2.8% at 10 years and 6.2% at 15 years in a cohort of 1,483 adult ALL patients. [15] On the other hand, several large cohort studies on child ALL have been published, with the largest number of 9,720 patients [14] and longest follow-up time of up to 30 years [11], and including a cumulative incidence of SN at 1.18–10.85% at 10–30 years of follow-up [8–14]. When compared with general population, the risk of SN increased significantly in child ALL (SIR 6.06) but not in the adult group (SIR 1.16), which suggests that the SN risk imposed by ALL and associated treatments seems to be more prominent in child ALL than in adult ALL.

CNS tumors were the most common SN in our study, which is consistent with the previous reports [10, 11, 13]. The mean latency time of developing CNS tumors was as long as 7.4 years in child ALL. This is similar to the finding that high-grade CNS tumors develop at nine years and low-grade CNS tumors at 20 years [9, 13]. In addition, we found that all child ALL patients in our study who developed CNS tumors had received cranial irradiation. Walter *et al*. reported the use of cranial irradiation as a risk factor of developing CNS tumors, with a dose-dependent response [13]. Three out of four patients who developed CNS tumors were aged less than six years in our study. Being less than six years old has also been reported as a risk factor of developing high-grade CNS tumors [13]. Due to the potential risk of cranial irradiation and its limited benefits, it is not routinely used as prophylaxis nowadays. On the contrary, CNS tumors in adult ALL have rarely been reported, and only one CNS tumor developed in adult ALL in our study. This may be due to the decreased use of cranial irradiation and more maturation of brain tissue in adult ALL.

Radiotherapy was considered to increase SN by its cytotoxic effects [22, 23]. In Taiwan, prophylactic cranial irradiation was commonly given with 0.5 Gy twice daily for a total dose of 18 Gy and total body irradiation for a total dose of 12 to 15 Gy. In our study, patients who had received total body irradiation and/or cranial irradiation were at risk of SN development according to the univariate analysis, and only cranial irradiation was an independent risk factor in the multivariate analysis. These results support those of previous reports, which have shown that irradiation increases the SN risk in child ALL [11] and that the risk is increased with higher radiation dose [10].

Chemotherapy agents have also been identified as increases in the risk of secondary leukemia and myelodysplastic syndrome in different kinds of primary cancers [24]. In child ALL studies, anthracycline, cyclophosphamide [10], 6-MP and methotrexate[8] have been shown to be independent risk factors for SNs, with secondary leukemia and myelodysplastic syndrome being the most common. However, we did not find a specific chemotherapy agent as a risk factor for SNs in our study. This may be because we did not include hematologic malignancies as SNs in our study.

Of note, the distribution of comorbidities was quite different between adults and child patients, especially comorbidities of COPD (15.6% v. 3.0%) and ESRD (2.3% v. 0.1%). We found ESRD was a significantly risk factor for SNs development only in adult ALL survivors. The underlying mechanism remained speculative. In a large cohort study, patients with ESRD was reported to have a higher incidence of developing cancers when compared to general population[25]. The possible mechanisms may relate to chronic infection, relatively immunocompromised, poor nutrition and underlying diseases that associated with ESRD, such as metabolic syndrome[26].

The SNs in adult ALL in our study included cancers of the breasts (3), head and neck (2), lung and mediastinum (1), esophagus (1), uterus (1) and bladder (1). These secondary solid organ neoplasms were quite different from those in Tavernier *et al*. [15], in which skin tumors (5), chondrosarcoma (1), thymoma (1), hisocytosis (1), tongue cancer (1), and bronchial cancer (1) were shown. Unlike Tavernier's study, which enrolled patients in two randomized trials (LALA-87 and LALA-94), our study comprises patients with a nationwide population-based non-selective enrollment and reflects a real-world situation in Taiwan. Based on the inconsistency of patient enrollments, the treatment policies differed; only 14% of the patients in our study received stem cell transplantation, while 33% patients in Tavernier's received it. A similar finding was that these SNs had a long latency. For skin tumors and other solid tumors, the latency time were 6.9 years and 3.7 years, respectively. It implies that the longer the survival of ALL patients, the more attention should be paid to the occurrence of SNs.

There are some limitations in our study. First, we did not have information about ALL risk groups, including immunophenotype, cytogenetics, and the laboratory data. Those higher-risk patients may receive more intensive treatments and thus have more possibility to have treatment-related cancer [10]. However, higher-risk patients have poorer survival, which makes it difficult to observe SNs. Hence, the relationship between risk group and SN is not clear at present. Second, we did not analyze the irradiation dose and subsequent risk of SNs, so a dose-response relationship is not available. Third, the cause of death and the disease status, either ALL or SN, were not part of the registry in the NHIRD database. Therefore, it is difficult to evaluate the impact of SN on the lifespan of ALL patients. Finally, because of the restrictions of our databases, we did not analyze the personal, environmental and family history information that may have effects on the development of SNs.

## Conclusions

In conclusion, this nationwide population-based study shows an increased risk in child ALL, and the risk increases with longer follow-up time. We also found that CNS tumors are the most common type of SNs, and that cranial irradiation is an independent risk factor. These findings are consistent with previous data, which supports a conservative strategy towards cranial irradiation for ALL patients. With the improving survival in ALL patients, we may need to be more aware of the early symptoms of developing of SNs, especially in those long-term survivors with risk factors.

## Supporting Information

S1 Table Standardized incidence ratios according to sex, age at diagnosis and duration of acute lymphoblastic leukemia (hematopoietic stem cell transplantation were censored). (DOCX)

S2 Table Standardized incidence ratios for secondary neoplasms, according to cancer site (hematopoietic stem cell transplantation were censored). (DOCX)

S3 Table Risk factors for secondary neoplasm development in patients with acute lymphoblastic leukemia (hematopoietic stem cell transplantation were censored). (DOCX)

S4 Table Risk factors for secondary neoplasm development in patients with acute lymphoblastic leukemia (Age ≥ 20). (DOCX)

S5 Table Risk factors for secondary neoplasm development in patients with acute lymphoblastic leukemia (Age < 20). (DOCX)
