# Correction: Secondary Solid Organ Neoplasm in Patients with Acute Lymphoblastic Leukemia: A Nationwide Population-Based Study in Taiwan

**DOI:** 10.1371/journal.pone.0162225

**Published:** 2016-08-25

**Authors:** Chung-Jen Teng, Leh-Kiong Huon, Yu-Wen Hu, Chiu-Mei Yeh, Sheng-Hsuan Chien, San-Chi Chen, Chia-Jen Liu

The Funding section appears incorrectly in the published article. The correct Funding statement is: This study was supported by grants from Taipei Veterans General Hospital (V105B-016 and V105E10-002-MY2-1), the Ministry of Science and Technology (MOST 104-2314-B-075-085-MY2), Taiwan Clinical Oncology Research Foundation, Szu-Yuan Research Foundation of Internal Medicine, and Chong Hin Loon Memorial Cancer and Biotherapy Research Center, at National Yang-Ming University.
