# Supplementary material for: Secondary Solid Organ Neoplasm in Patients with Acute Lymphoblastic Leukemia: A Nationwide Population-Based Study in Taiwan
Source: PLoS One. 2016 Apr 1;11(4):e0152909. doi: 10.1371/journal.pone.0152909 (PMC4817987; doi:10.1371/journal.pone.0152909)
Supplement: S1 Table — (DOCX) [file pone.0152909.s001.docx]

**S1 Table Standardized incidence ratios according to sex, age at diagnosis and duration of acute lymphoblastic leukemia (hematopoietic stem cell transplantation were censored)**

|  | Total |  |  |  |  |
| --- | --- | --- | --- | --- | --- |
| Characteristics | Observed | Expected | SIR (95% CI) |  |  |
| All cancers | 10 | 8.18 | 1.22 (0.59–2.25) |  |  |
| Age at diagnosis, years |  |  |  |  |  |
| 0–19 | 3 | 0.79 | 3.78 (0.78–11.06) |  |  |
| 20–39 | 3 | 1.14 | 2.63 (0.54–7.68) |  |  |
| ≥ 40 | 4 | 6.24 | 0.64 (0.20–1.55) |  |  |
| Follow-up time period after acute lymphoblastic leukemia | | | |  |  |
| 0–1 | 2 | 2.67 | 0.75 (0.09–2.71) |  |  |
| 1–5 | 0 | 2.86 | 0.00 (0.00–1.29) |  |  |
| 5–10 | 3 | 1.84 | 1.63 (0.34–4.77) |  |  |
| ≥ 10 | 5 | 0.81 | 6.15 (2.00–14.34) |  |  |

Abbreviations: SIR, standardized incidence ratio; CI, confidence interval
