# Supplementary material for: Secondary Solid Organ Neoplasm in Patients with Acute Lymphoblastic Leukemia: A Nationwide Population-Based Study in Taiwan
Source: PLoS One. 2016 Apr 1;11(4):e0152909. doi: 10.1371/journal.pone.0152909 (PMC4817987; doi:10.1371/journal.pone.0152909)
Supplement: S2 Table — (DOCX) [file pone.0152909.s002.docx]

**S2 Table Standardized incidence ratios for secondary neoplasms, according to cancer site (hematopoietic stem cell transplantation were censored)**

|  | Total |  |  |  |
| --- | --- | --- | --- | --- |
| Site of cancer | Observed | Expected | SIR (95% CI) |  |
| All cancers | 10 | 8.18 | 1.22 (0.59–2.25) |  |
| Head and neck | 1 | 0.83 | 1.20 (0.03–6.69) |  |
| Esophagus | 0 | 0.14 | 0.00 (0.00–25.79) |  |
| Liver and biliary tract | 1 | 1.06 | 0.94 (0.02–5.24) |  |
| Lung and mediastinum | 0 | 0.82 | 0.00 (0.00–4.47) |  |
| Breast | 3 | 1.10 | 2.74 (0.56–8.00) |  |
| Uterus | 1 | 0.16 | 6.08 (0.15–33.88) |  |
| Bladder | 1 | 0.21 | 4.77 (0.12–26.60) |  |
| CNS | 3 | 0.40 | 7.55 (1.56–22.07) |  |

Abbreviations: SIR, standardized incidence ratio; CI, confidence interval; CNS, central nervous system
