# Supplementary material for: Secondary Solid Organ Neoplasm in Patients with Acute Lymphoblastic Leukemia: A Nationwide Population-Based Study in Taiwan
Source: PLoS One. 2016 Apr 1;11(4):e0152909. doi: 10.1371/journal.pone.0152909 (PMC4817987; doi:10.1371/journal.pone.0152909)
Supplement: S3 Table — (DOCX) [file pone.0152909.s003.docx]

**S3 Table Risk factors for secondary neoplasm development in patients with acute lymphoblastic leukemia (hematopoietic stem cell transplantation were censored)**

|  | Univariate analysis | |  | Multivariate analysis^a^ | |
| --- | --- | --- | --- | --- | --- |
| Variables | HR (95% CI) | *P* Value |  | HR (95% CI) | *P* Value |
| Age ≥ 20 | 8.90 (2.42–32.78) | 0.001 |  | 8.14 (1.69–39.25) | 0.009 |
| Sex (male) | 0.35 (0.09–1.34) | 0.124 |  |  |  |
| **Comorbidities** |  |  |  |  |  |
| Diabetes mellitus | **†** |  |  |  |  |
| Chronic pulmonary disease | 13.02 (2.40–70.54) | 0.003 |  | 3.76 (0.61–23.12) | 0.153 |
| ESRD | 51.41 (5.62–470.00) | 0.001 |  | 56.03 (3.28–956.39) | 0.005 |
| Cirrhosis | **†** |  |  |  |  |
| Autoimmune diseases | **†** |  |  |  |  |
| Dyslipidemia | **†** |  |  |  |  |
| **Treatment^b^** |  |  |  |  |  |
| Anthracyclines | 0.48 (0.12–1.87) | 0.289 |  |  |  |
| Akylating agents | 1.39 (0.39–4.90) | 0.610 |  |  |  |
| Antimetabolites | 0.82 (0.17–3.91) | 0.803 |  |  |  |
| Topo-II inhibitor | 0.41 (0.10–1.60) | 0.198 |  |  |  |
| Asparaginase | 0.34 (0.09–1.20) | 0.094 |  | 0.24 (0.05–1.21) | 0.084 |
| Cranial irradiation | 5.71 (1.31–24.85) | 0.020 |  | 21.12 (3.61–123.41) | 0.001 |

Abbreviations: ESRD, end-stage renal disease; Topo, topoisomerase; TBI, total body irradiation; HSCT, hematopoietic stem cell transplantation

^a^All factors with *p* < 0.1 in univariate analyses were included in the Cox multivariate analysis.

^b^Treatment was analyzed as a time-dependent covariate in the Cox regression model.

**†**: Don’t converge.
