# Supplementary material for: Secondary Solid Organ Neoplasm in Patients with Acute Lymphoblastic Leukemia: A Nationwide Population-Based Study in Taiwan
Source: PLoS One. 2016 Apr 1;11(4):e0152909. doi: 10.1371/journal.pone.0152909 (PMC4817987; doi:10.1371/journal.pone.0152909)
Supplement: S4 Table — (DOCX) [file pone.0152909.s004.docx]

**S4 Table Risk factors for secondary neoplasm development in patients with acute lymphoblastic leukemia (Age ≥ 20)**

|  | Univariate analysis | |  | Multivariate analysis^a^ | |
| --- | --- | --- | --- | --- | --- |
| Variables | HR (95% CI) | *P* Value |  | HR (95% CI) | *P* Value |
| Sex (male) | 0.52 (0.13–2.09) | 0.355 |  |  |  |
| **Comorbidities** |  |  |  |  |  |
| Diabetes mellitus | **†** |  |  |  |  |
| Chronic pulmonary disease | 3.91 (0.76–20.01) | 0.102 |  |  |  |
| ESRD | 20.31 (2.07–199.51) | 0.010 |  | 34.56 (3.01–396.56) | 0.004 |
| Cirrhosis | **†** |  |  |  |  |
| Autoimmune diseases | **†** |  |  |  |  |
| Dyslipidemia | **†** |  |  |  |  |
| **Treatment^b^** |  |  |  |  |  |
| Anthracyclines | 0.98 (0.22–4.25) | 0.974 |  |  |  |
| Akylating agents | 1.20 (0.30–4.82) | 0.793 |  |  |  |
| Antimetabolites | 2.61 (0.51–13.39) | 0.249 |  |  |  |
| Topo-II inhibitor | 1.87 (0.43–8.05) | 0.401 |  |  |  |
| Asparaginase | 1.08 (0.28–4.22) | 0.914 |  |  |  |
| Cranial irradiation | 5.24 (1.28–21.37) | 0.021 |  | 6.38 (1.43–28.44) | 0.015 |
| TBI | 2.19 (0.41–11.63) | 0.356 |  |  |  |
| HSCT | 2.34 (0.52–10.61) | 0.270 |  |  |  |

Abbreviations: COPD, chronic obstructive pulmonary disease; ESRD, end-stage renal disease; Topo, topoisomerase; TBI, total body irradiation; HSCT, hematopoietic stem cell transplantation

^a^All factors with *p* < .1 in univariate analyses were included in the Cox multivariate analysis.

^b^Treatment was analyzed as a time-dependent covariate in the Cox regression model.

**†**: Don’t converge.
