# Supplementary material for: Secondary Solid Organ Neoplasm in Patients with Acute Lymphoblastic Leukemia: A Nationwide Population-Based Study in Taiwan
Source: PLoS One. 2016 Apr 1;11(4):e0152909. doi: 10.1371/journal.pone.0152909 (PMC4817987; doi:10.1371/journal.pone.0152909)
Supplement: S5 Table — (DOCX) [file pone.0152909.s005.docx]

**S5 Table Risk factors for secondary neoplasm development in patients with acute lymphoblastic leukemia (Age < 20)**

|  | Univariate analysis | |  | Multivariate analysis^a^ | |
| --- | --- | --- | --- | --- | --- |
| Variables | HR (95% CI) | *P* Value |  | HR (95% CI) | *P* Value |
| Sex (male) | 0.70 (0.25–1.93) | 0.490 |  |  |  |
| **Comorbidities** |  |  |  |  |  |
| Diabetes mellitus | **†** |  |  |  |  |
| Chronic pulmonary disease | 5.59 (1.21–25.88) | 0.028 |  | **†** |  |
| ESRD | 23.33 (2.96–184.02) | 0.003 |  | **†** |  |
| Cirrhosis | **†** |  |  |  |  |
| Autoimmune diseases | **†** |  |  |  |  |
| Dyslipidemia | **†** |  |  |  |  |
| **Treatment^b^** |  |  |  |  |  |
| Anthracyclines | **†** |  |  |  |  |
| Akylating agents | 4.65 (0.54–39.81) | 0.161 |  |  |  |
| Antimetabolites | **†** |  |  |  |  |
| Topo-II inhibitor | 2.76 (0.31–24.66) | 0.364 |  |  |  |
| Asparaginase | 0.66 (0.08–5.71) | 0.705 |  |  |  |
| Cranial irradiation | 8.59 (0.99–74.35) | 0.051 |  | 7.22 (0.82–63.20) | 0.074 |
| TBI | 4.20 (0.49–35.98) | 0.191 |  |  |  |
| HSCT | 8.40 (1.54–45.89) | 0.014 |  | 6.67 (1.21–36.88) | 0.030 |

Abbreviations: COPD, chronic obstructive pulmonary disease; ESRD, end-stage renal disease; Topo, topoisomerase; TBI, total body irradiation; HSCT, hematopoietic stem cell transplantation

^a^All factors with *p* < .1 in univariate analyses were included in the Cox multivariate analysis.

^b^Treatment was analyzed as a time-dependent covariate in the Cox regression model.

**†**: Don’t converge.
